# A novel approach combining YOLO and DeepSORT for detecting and counting live fish in natural environments through video

**DOI:** 10.1371/journal.pone.0323547

**Published:** 2025-06-11

**Authors:** Nguyen Minh Khiem, Tran Van Thanh, Nguyen Hung Dung, Yuki Takahashi

**Affiliations:** 1 College of Information & Communication Technology, Can Tho University, Can Tho, Vietnam; 2 FPT University, Can Tho, Vietnam; 3 Faculty of Fisheries Sciences, Hokkaido University, Hakodate, Hokkaido, Japan; Bigelow Laboratory for Ocean Sciences, UNITED STATES OF AMERICA

## Abstract

Applying Artificial Intelligence (AI) to the monitoring of live fish in natural environments represents a promising approach to the sustainable management of aquatic resources. Detecting and counting fish in water through video analysis is crucial for fish population statistics. This study employs AI algorithms, specifically YOLOv10 (You Only Look Once version 10) for identifying the presence fish in video frames, combined with the DeepSORT (Deep Simple Online and Realtime Tracking) algorithm to count the number of fish individual moving across the frames. A total of 9,002 frames were extracted from 13 videos recorded in five different environments: areas with submerged tree roots, shallow marine regions, coral reefs, bleached coral reefs and seagrass meadows. To train the recognition model, the dataset was divided into training, validation and testing sets in 8:1:1 ratio. The results demonstrated that the model achieved an accuracy of 89.5%, with processing times of 6.2ms for preprocessing, 387.0ms for inference and 0.9ms for postprocessing per image. The combination of YOLO and DeepSORT enhances the accuracy of tracking objects in aquatic environments, showing great potential for the monitoring of fishery resources.

## Introduction

Accurately monitoring information on the presence and abundance of fish in natural environments is fundamental to the conservation of aquatic resources. This enables effective management of fish density, the observation of their behaviors, and a deeper understanding of their habitats [1]. Identifying the presence of fish and tracking them in real-time underwater is an effective approach for estimating fish population status, preventing overfishing, and supporting biodiversity assessment and the conservation of aquatic species [2]. Additionally, information on fish density provides critical data for predicting environmental fluctuations and fostering the development of sustainable ecosystems.

Monitoring fish in the wild, particularly in marine environments, faces numerous challenges and difficulties. This task requires observation across diverse habitats, and the vast volume of image data involved makes manual processing both time-consuming and costly [3]. Fish tracking has traditionally been done manually, which is often inefficient and prone to errors. It requires expert involvement, and sending images to ichthyologists for analysis can reduce image quality, resulting in inaccurate data and a lack of real-time continuous monitoring [4]. Additionally, image processing in monitoring requires high levels of automation and accuracy, as images are continuously transmitted and change over time. Automated processing is not affected by human bias or fatigue, leading to more reliable and valuable data. This necessity drives the integration of artificial intelligence, particularly in image analysis and processing, into monitoring systems.

Recent studies have shown that the use of computer vision in fisheries has proven highly valuable for statistical analysis and resource management. The development of fish tracking algorithms plays a crucial role in observing fish behavior [5], determining fish population density [6], and reducing the need for divers or manual observation which can be time-consuming and low accuracy. Additionally, computer vision techniques are non-invasive, helping to minimize stress on fish and preserve their natural behaviors.

The implementation of the YOLO (You Only Look Once) algorithm in fisheries presents significant promise and potential for overcoming various monitoring challenges due to its exceptional ability to perform rapid object detection, thereby facilitating real-time image and video processing [7]. Unlike traditional methodologies that segment input images into smaller regions for individual processing, YOLO processes the entire image in a single pass, directly predicting bounding boxes and associated class probabilities. This approach markedly reduces processing time while substantially enhancing detection speed [8]. Recent research has validated the high efficacy of YOLO in agricultural applications, including the determination of fruit ripeness and automated harvesting, as demonstrated with tomatoes [9], as well as in estimating the weight and ripeness of oranges [10]. In the transportation sector, YOLO has been effectively employed for the recognition of general unmanned aerial vehicles [11] and the detection of pedestrians, cars, buses, and motorcycles [12]. In the field of fisheries, various versions of YOLO have been utilized for detecting the presence of whiteleg shrimp, *Litopenaeus vannamei* [13], identifying fish species in dynamic underwater environments [14], and recognizing different fish species [15].

To track moving objects in video, DeepSORT (Deep Simple Online and Realtime Tracking) is a viable choice due to its advanced capabilities in object tracking. This algorithm overcomes the limitations of traditional tracking techniques, such as occlusions, rapid object movement and varying lighting conditions [16]. In fisheries, DeepSORT provides highly accurate tracking results by combining appearance features with traditional Kalman filtering and the Hungarian assignment algorithm [17]. This combination allows for precise object tracking even when objects temporarily disappear or overlap [18]. Additionally, DeepSORT is designed for real-time operation, making it well-suited for applications in fisheries where immediate fish tracking is necessary for tasks such as population monitoring, behavior analysis or regulatory compliance. The algorithm effectively handles changes in fish appearance caused by variations in lighting, viewing angles, and movement direction, which is crucial in dynamic aquatic environments. Recently, DeepSORT has been used to measure shrimp length and track swimming speed [19]. A study [20] demonstrated that combining deep learning models with DeepSORT significantly enhances the ability to track the movement of species in agricultural and fisheries contexts.

To address the challenges in monitoring and leverage the advancements of AI in fishery management, in this study, we used YOLOv10 to train a model that detects fish in video frames captured from marine environment. We labeled each frame with the locations of the detected fish, while areas where no fish are found remain unlabeled. To count the fish, we combined YOLO with the DeepSORT algorithm, which helps identify and track individual fish across all the frames in the video. We also explained the steps in our process to show how each algorithm works and how we evaluate the accuracy of our results.

## Materials and methods

### Preliminaries

**Fig 1:**
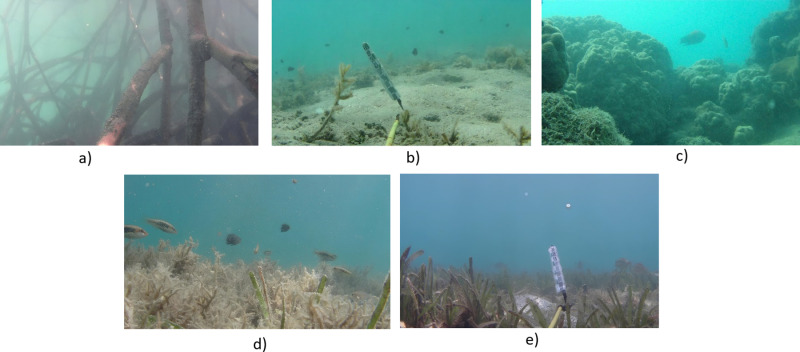
The fish habitat environments for recoding include: (a) areas with tree roots submerged in water, (b) shallow coastal zones, (c) coral reefs, (d) bleached coral reefs, and (e) seagrass meadows.

The fish detect dataset comprises 9,002 images, each representing a frame extracted from 13 recorded videos using OpenCV in Python. These videos were collected from FishTrac dataset [21], where they are publicly available and have been published to support research and development in marine environment monitoring. These frames, captured at 24 frames per second, have a resolution of 1,920x1,080 pixels and were captured in various water environments, including (1) areas with submerged tree roots, (2) shallow marine regions, (3) coral reefs, (4) bleached coral reefs and (5) seagrass meadows as shown in Fig 1.

This dataset was divided into three parts: a training set (80%), a test set (10%) and a validation set (10%). The division was performed across the corresponding environments, as detailed in the Table 1.

**Table 1:** The number of images in training, testing, and validation sets corresponds to the five aquatic environments.

Environment	Flooded root zones	Shallow coastal zones	Coral reefs	Bleached coral reefs	Seagrass meadows
Trainning set	2,688	946	2,028	720	802
Testing set	340	120	258	90	101
Validation set	340	120	258	90	101
Total	3,368	1,186	2,544	900	1,004

**Fig 2:**
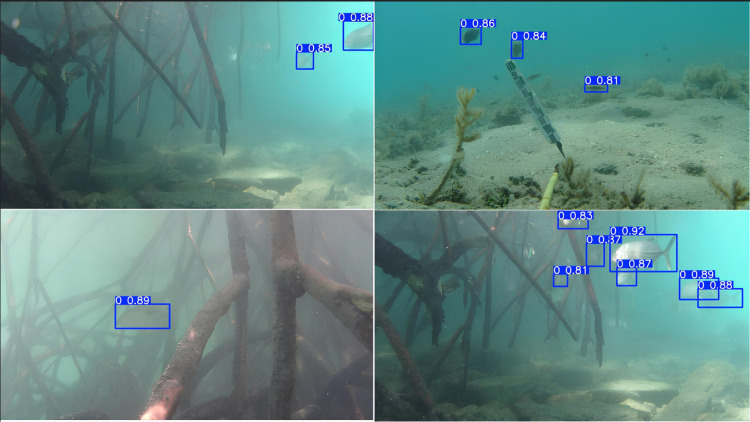
Label indicating the presence of fish in video frames, with each bounding box containing an individual fish.

Each image is paired with a corresponding label file. These label files provide detailed information about the position of fish present in the image. Specifically, they contain the coordinates of the bounding boxes that enclose the fish within each image, along with the corresponding class labels for these fish species. It’s important to note that the bounding box annotations are normalized according to the image size, with values ranging from 0 to 1. Additionally, there is only one label indicating whether fish are present or not, as shown in Fig 2.

**Fig 3:**
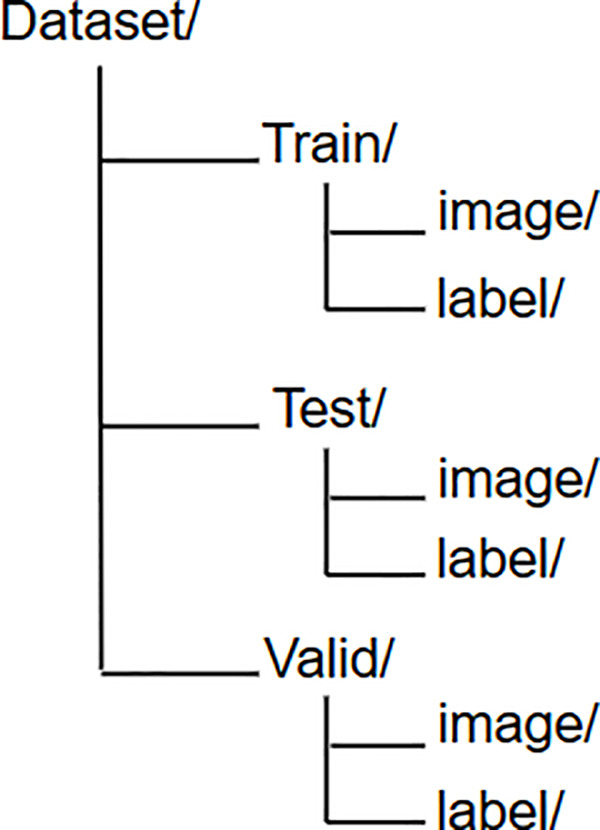
The data storage structure includes separate folders for Training, Validation, and Test sets. Each folder contains images and their corresponding labels.

To ensure that each image has a corresponding label file, which facilitates accurate and efficient model training, testing, and validation, the data for each file is organized into folders as in Fig 3.

### Model for fish detecting

#### YOLOv10.

The technical background of YOLOv10 is rooted in the development of the YOLO family of object detection models. YOLOv10 builds upon the advancements and enhancements of its predecessors, particularly YOLOv5, YOLOv6, and YOLOv8 [22]. YOLOv10 have introduced significant advancements over previous versions by incorporating transformer layers into its architecture. This enhancement improves the model’s ability to capture global context and long-term relationships within images, which is crucial for detecting small objects and handling complex scenes. YOLOv10 continues to leverage Feature Pyramid Networks (FPNs), a technique for multi-scale feature extraction, which enhances the model’s ability to detect objects at various sizes. The introduction of advanced FPN variants, such as the Path Aggregation Network (PAN), further boosts this capability [23].

In this study, we utilized a GPU for the training process, with the “device” parameter set to “cuda:0” to accelerate model training. Additionally, we employed parallel threads with the “workers” parameter set to 8, ensuring efficient data loading and processing for uninterrupted model training.

#### DeepSORT.

DeepSORT is an advanced techsnique in computer vision and object tracking [16]. It utilizes deep neural networks to extract features from detected objects. Based on these features, DeepSORT calculates the similarity between detections, which is crucial for identifying objects across consecutive frames in a video. The DeepSORT algorithm consists of several steps. First, it begins with object detection in each frame using an external detection model that provides bounding boxes for the identified objects. To uniquely distinguish these objects across frames, DeepSORT extracts deep appearance features from each detected object using a pre-trained convolutional neural network. These features, combined with spatial information, enable robust data association, which is processed by a Kalman filter to predict the positions of objects in subsequent frames and the Hungarian algorithm to assign new detections to existing tracks.

DeepSORT also incorporates a matching cascade strategy to prioritize recently observed tracks, reducing the likelihood of identity swaps. By combining appearance-based re-identification with traditional tracking methods, DeepSORT maintains accurate and consistent tracking of multiple objects, even in challenging situations such as occlusions or temporary departures and returns of objects within the frame.

During real-time object tracking with the DeepSORT algorithm, the parameter max_age (the maximum lifetime of a track) was set to 60, allowing objects to persist for up to 60 frames without a new detection before being removed. This helps minimize the early elimination of temporarily lost objects. The n_init parameter, which specifies the number of detections required to confirm a track, was set to 8, meaning a new track must be detected at least 8 times consecutively to be validated. This parameter helps filter out unstable or false tracks, ensuring that only objects showing stable evidence are tracked. The min_confidence parameter, which sets the minimum confidence threshold for a detection, was adjusted to eliminate low-confidence detections, thereby improving the system’s accuracy. Other parameters such as max_iou_distance, which controls the accuracy of linking closely moving objects, nn_budget, which efficiently manages computational resources as the number of tracked objects increases, and use_cuda, which ensures efficient real-time operation, were utilized with their default values.

#### Detecting and Tracking fish.

The combination of YOLO with DeepSORT for tracking fish in video frames was implemented through the following steps:

- Input Image/Video: The process started with input images or videos that contain the fish objects to be tracked.
- Object Detection (YOLOv10): YOLOv10 detected fish objects within the images or video frames, generating bounding boxes and class labels for each detected object.
- Feature Extraction: A deep learning network was used to extract features from the detected objects.
- Object Association (DeepSORT): DeepSORT applied an association algorithm to identify and track the objects across frames based on the extracted features.
- Position Prediction (Kalman Filter): The Kalman filter was used to predict the future positions of the objects and smooth their movements.
- Output: The final output includes the positions and tracking information of the objects across the video frames.

**Fig 4:**
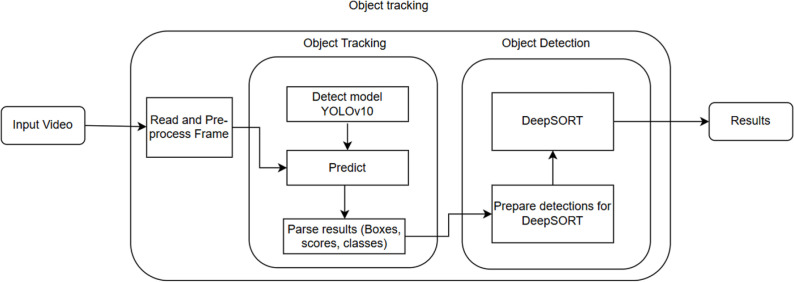
Flow of tracking fish: The video is input through ‘Read and Preprocess Frame’. YOLOv10 predicts boxes, scores, and classes for each frame, which are then fed into DeepSort for tracking. The final output is a video containing the tracked fish objects across all frames.

These steps are summarized as in Fig 4.

#### Counting fish.

**Fig 5:**
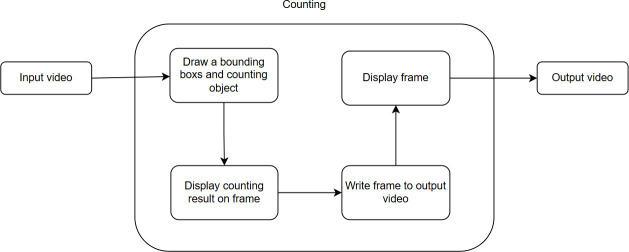
Flow of counting fish: The input video is processed to draw bounding boxes around fish and count the individuals. The counting result is displayed on each frame, and the frames are then written to the output video, which is displayed as the final result.

Counting the number of fish based on the outputs from YOLO and DeepSORT involves determining the quantity of fish in each frame and tracking this count across video frames. This is useful for monitoring and studying fish populations in natural environments. The counting process is illustrated in Fig 5.

## Results

The input video used to test the model was a short clip capturing underwater scenes with various fish species in motion. This video contains high-resolution frames, ensuring data quality sufficient for evaluating the accuracy of the detection model. The video had a duration of 13 seconds and includes a total of 676 frames.

**Fig 6:**
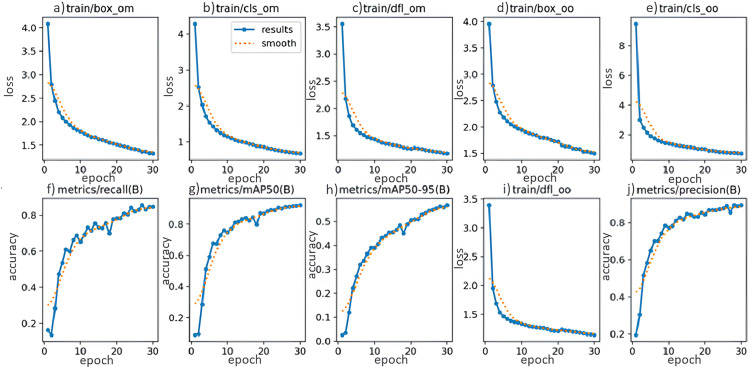
Result of training model. Note that, cls_om represents the Classification Object Model loss, dfl_om denotes the Distance Focal Object Model loss, and train/box_oo refers to the Bounding Box Object Optimization loss.

In this experiment, the dataset was utilized to train the YOLOv10 model for 30 epochs, allowing the model adequate time to learn and distinguish fish in different scenes while mitigating the risk of overfitting. The results, as shown in the figure below, demonstrate a significant performance improvement with a mAP50 value, meaning Mean Average Precision, is a performance metric for object detection models, reaching 89.5% which is shown as in Fig 6.

During the training of the YOLO model, evaluation metrics for accuracy and loss were monitored and recorded for each epoch, as shown in Fig 6. Specifically, the charts for box loss and classification loss for both training datasets, “om” and “oo” demonstrate a gradual reduction over the epochs, indicating that the model is learning effectively and optimizing over time. Additionally, the Distance Focal Loss, which measures prediction deviation, also shows a decreasing trend, reflecting the model’s improving accuracy in object localization.

Furthermore, performance metrics such as recall, precision, and Mean Average Precision (mAP) increased over time. Notably, mAP50, which measures average precision with a 50% IoU threshold, and mAP50-95, which measures precision across IoU thresholds ranging from 50% to 95%, both showed significant improvements. This indicates that the model’s object detection capabilities are enhancing. The continuous increase in these metrics across epochs confirms that the YOLO model is learning effectively, achieving substantial improvements in accuracy and generalization for objects in the test dataset.

Overall, these results demonstrate that the YOLO model not only minimizes errors during training but also enhances object detection performance, affirming its potential for widespread application in computer vision tasks.

### Detecting fish by YOLOv10

**Fig 7:**
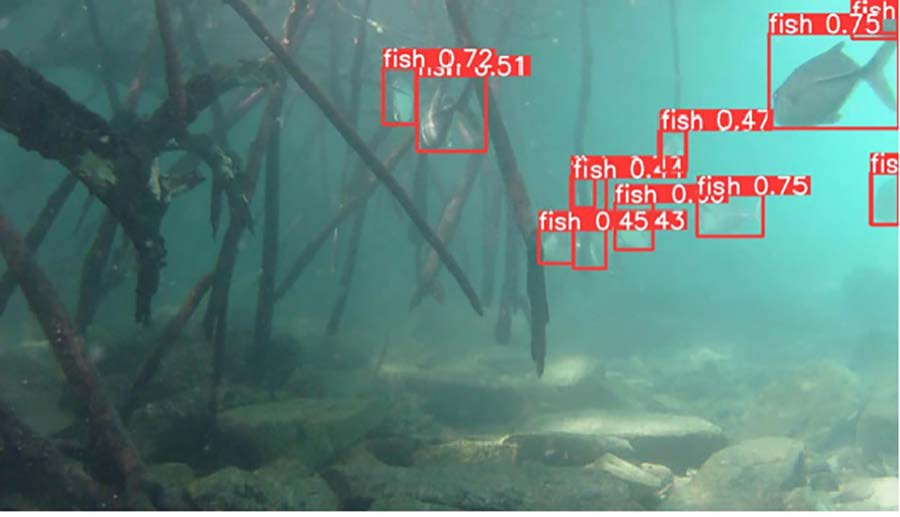
Detection of Fish in Areas with Tree Roots Submerged in Water.

**Fig 8:**
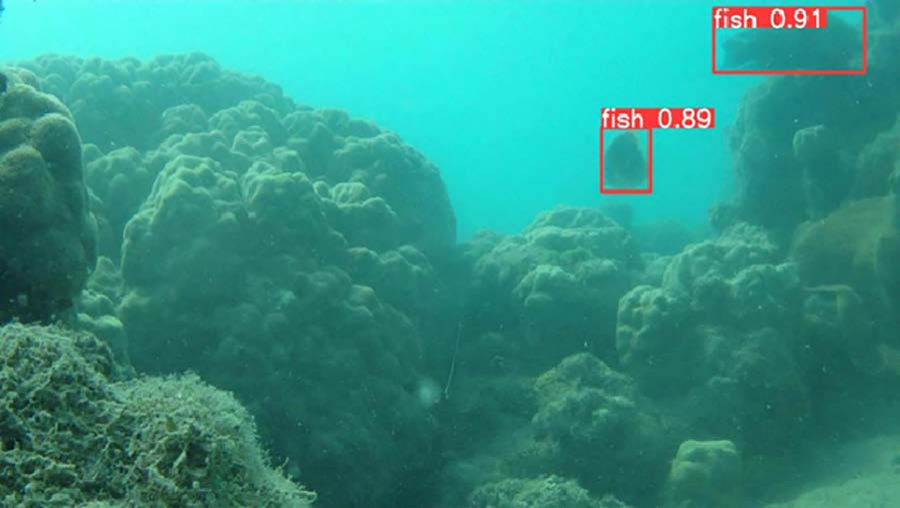
Detection of fish in shallow coastal zones.

**Fig 9:**
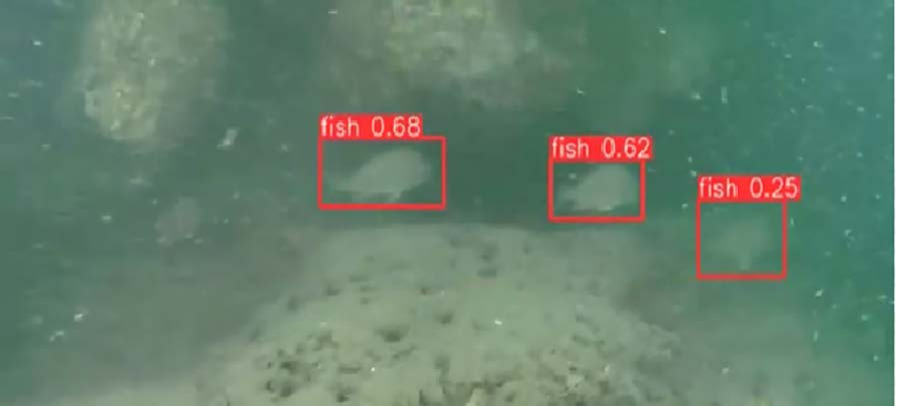
Detection of Fish in coral reefs.

**Fig 10:**
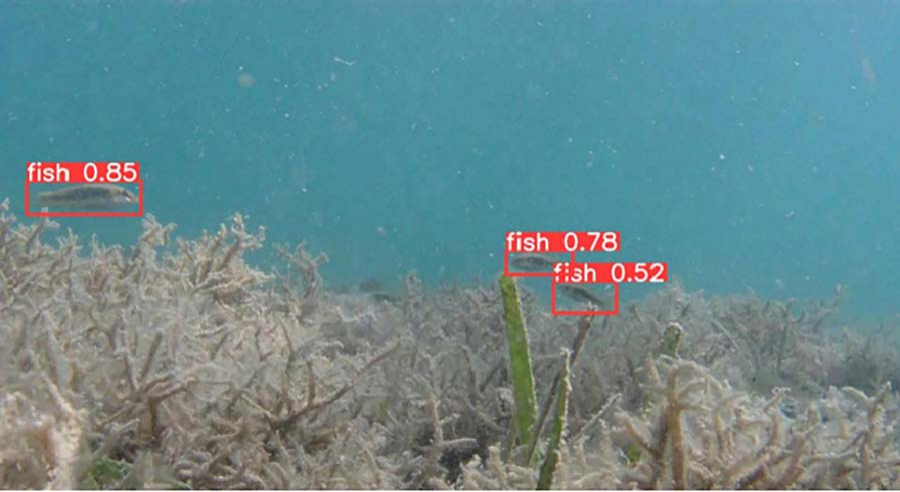
Detection of Fish in bleached coral reefs.

**Fig 11:**
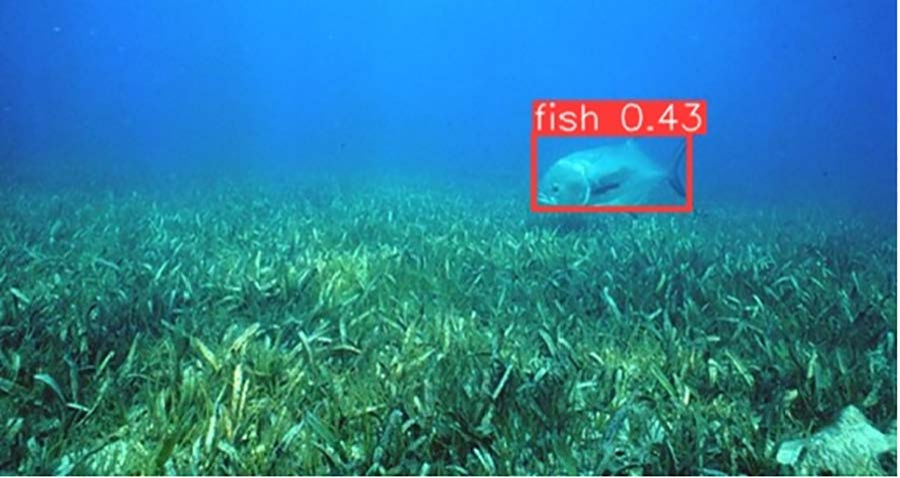
Detection of Fish in seagrass meadows.

The output file from YOLOv10 includes frames from the input video with bounding boxes drawn around the detected fish objects. Each bounding box is assigned a class label and a corresponding confidence score, which facilitates easy verification and evaluation of the model’s accuracy. The results of determining the presence of individual fish with their corresponding confidence levels are illustrated in Figs 7–11, which correspond to the following five environments: areas with tree roots submerged in water, shallow coastal zones, coral reefs, bleached coral reefs, and seagrass meadows.

### Tracking and counting fish by YOLOv10 and DeepSORT

**Fig 12:**
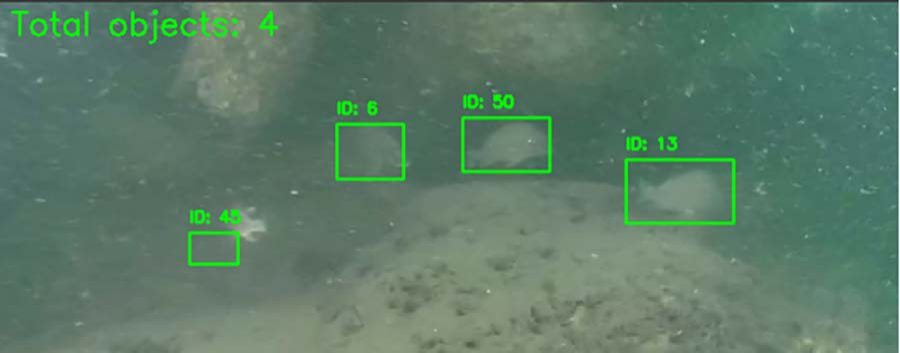
Fish counting results show the number of objects with bounding boxes in each frame.

After detecting the objects in the video, the DeepSORT algorithm is used to track these objects across frames. This allows us to assign IDs to each object from the moment they appear and monitor their movement through the video frames. In Fig 12, four fish objects are detected, with IDs 45, 6, 50, and 13.

## Discussion

In this study, we applied the YOLOv10 and DeepSORT algorithms to detect the presence of fish in aquatic environments and count the number of fish appearing in videos. The model was trained on a dataset collected from various environments, including areas with submerged tree roots, shallow coastal zones, coral reefs, bleached coral reefs, and seagrass meadows. The results demonstrate a high accuracy in counting, with a mAP50 of 89.5%.

The utilization of video data containing fish detection and tracking not only enables the automatic, rapid, and accurate estimation of fish populations but also enhances monitoring efficiency. Notably, with the support of this computer vision techniques, the system can precisely identify individual fish even when they are continuously moving and swimming in schools in real time. This capability significantly contributes to biological research and the effective management of aquatic resources.

YOLOv10 was chosen for this task due to its superior improvements over previous versions, providing significant results in image detection [24]. Specifically, YOLOv10 features an optimized network architecture with enhanced CNN layers. It is designed for fast object detection by reducing unnecessary parameters and computations, and it can detect and classify more complex and overlapping objects with diverse sizes and shapes. Consequently, YOLOv10 achieves higher accuracy in object detection, particularly in challenging conditions such as poor lighting or partial occlusion, as seen in the varied environments of the dataset.

The combination of YOLO with DeepSORT for tracking and counting fish in videos yields accurate results because YOLO handles object detection at high speeds, while DeepSORT maintains continuous object tracking without compromising processing speed. Together, YOLO detects objects in each frame, and DeepSORT assigns IDs to these objects (Fig 12) and tracks them across consecutive frames. This improves the accuracy of object tracking, even in cases of occlusion or changing viewpoints, by using the Kalman Filter to predict the next position of the objects. This ensures precise tracking of fish even in low-light or partially obscured environments.

Furthermore, this study demonstrates that combining YOLOv10—an advanced version of image recognition algorithms—with motion-tracking algorithms like DeepSORT is promising and valuable in the field of aquaculture. Previous research has explored the precise detection capabilities of YOLO combined with DeepSORT’s tracking ability to enhance the accuracy of monitoring aquatic species’ activities. Our research reaffirms the suitability and potential of this approach for monitoring fish movements in aquatic environments.

In the future, we plan to apply this algorithm to track specific swimming behaviors or feeding patterns of aquatic species. In aquaculture, this will be effective for diagnosing diseases through monitoring swimming abnormalities or detecting harmful predators without causing stress to the fish during observation. In natural environments, this tracking method, combined with predictive capabilities and new species detection, holds great promise for future applications.

## Acknowledgment

This work was partially funded and supported by JSPS J-PEAKS.
